# Radiation-Induced Leiomyosarcoma: Does Antimetabolite Chemotherapy Contribute? A Report of Three Cases

**DOI:** 10.1080/13577140310001650330

**Published:** 2003

**Authors:** Bruce Brockstein, Arno Mundt, Daniel J. Haraf, Mark Ferguson, Anthony Montag

**Affiliations:** 1 Department of Medicine Northwestern University Evanston Northwestern Healthcare 2650 Ridge Ave. Evanston IL 60201 USA; 2 Department of Radiation and Cellular Oncology University of Chicago Chicago IL USA; 3 Department of Surgery University of Chicago Chicago IL USA; 4 Department of Pathology University of Chicago Chicago IL USA

## Abstract

*Purpose:* Radiation therapy in low and high doses is known to be associated with the occurrence of late secondary sarcomas. The addition of chemotherapy has not been clearly demonstrated as a contributing factor. We describe three patients with radiation-associated leiomyosarcoma who had also received antimetabolite chemotherapy.

*Methods:* Three cases of leiomyosarcoma occurring 9–27 years after radiation and antimetabolite chemotherapy are presented, along with histopathological details. A Medline search was used to assess prior reports of leiomyosarcoma after radiation.

*Results:* These three cases appear to be the first reported in which leiomyosarcoma followed therapy with radiation and antimetabolites.

*Discussion:* With the increasing use of antimetabolite therapy combined with radiation, there is the potential for more occurrences of leiomyosarcoma or other post-treatment sarcomas.


*Sarcoma*, September/December 2003, *VOL. 7, NO. 3/4, 167–172*

Taylor & Francis healthsciences

CASE REPORT

### Radiation-induced leiomyosarcoma: does antimetabolite chemotherapy contribute? A report of three cases

BRUCE BROCKSTEIN[1], ARNO MUNDT[2], DANIEL J. HARAF[2], MARK FERGUSON[3] & ANTHONY MONTAG[4]

[1]*Department of Medicine, Northwestern University, Evanston Northwestern Healthcare, Evanston, IL, USA, Department of* [2]*Radiation and Cellular Oncology,* [3]*Surgery &* [4]*Pathology, University of Chicago, Chicago, IL, USA*

**Abstract**

*Purpose:* Radiation therapy in low and high doses is known to be associated with the occurrence of late secondary sarcomas. The addition of chemotherapy has not been clearly demonstrated as a contributing factor. We describe three patients with radiation-associated leiomyosarcoma who had also received antimetabolite chemotherapy.

*Methods:* Three cases of leiomyosarcoma occurring 9–27 years after radiation and antimetabolite chemotherapy are presented, along with histopathological details. A Medline search was used to assess prior reports of leiomyosarcoma after radiation.

*Results*: These three cases appear to be the first reported in which leiomyosarcoma followed therapy with radiation and antimetabolites.

*Discussion:* With the increasing use of antimetabolite therapy combined with radiation, there is the potential for more occurrences of leiomyosarcoma or other post-treatment sarcomas.

**Key words:** *leiomyosarcoma, radiation, chemotherapy, antimetabolite, secondary malignancy*

### Introduction

There is a clear association between radiotherapy and an increased risk of development of secondary solid tumors, including sarcomas.[1–11] Patients treated with radiotherapy (RT) for Hodgkin's disease have a risk as high as 20% at 20 years, and much of this excess risk is attributable to radiotherapy with or without chemotherapy.[12,13] Therapeutic radiation for other malignant and benign diseases has also been associated with secondary malignancies, including secondary sarcomas.[14,17] Numerous histological subtypes of secondary sarcoma have been reported, but the most common have been osteosarcoma, fibrosarcoma, malignant fibrous histiocytoma (MFH), and angiosarcoma. Leiomyosarcoma (LMS) has been reported to occur after radiotherapy, but mostly in single case reports, and several very small series. These LMS have occurred in diverse sites, after 6–35 years, and after treatment for a variety of malignancies.[18–42]

In this report, we describe a series of three patients who developed leiomyosarcoma in the field of therapeutic radiation after 9–27 years. All had also been treated with antimetabolite chemotherapy.

### Patients and methods

We performed a Medline search encompassing the dates 1966–2001. Search terms were used to link radiation therapy, chemotherapy, antimetabolites, and Hodgkin's disease to secondary malignancies, solid tumors, sarcoma, and leiomyosarcoma. Reports of radiation-associated leiomyosarcoma were reviewed for elapsed time between RT and secondary leiomyosarcoma, as well as association of chemotherapy with RT. The cases are summarized in Table 1.

### Case reports and results

### *Case 1*

A 71-year-old man was treated in 1984 for rectal cancer with a low anterior resection and post-operative 5-fluorouracil, together with radiotherapy

Correspondence to: Bruce Brockstein, M.D., Assistant Professor of Medicine, Northwestern University, Evanston Northwestern Healthcare, 2650 Ridge Ave., Evanston, IL 60201, USA. Tel.: +1-847-570-1489; Fax: +1-847-570-2336; E-mail: b-brockstein@northwestern.edu

1357-714X print/1369–1643 online © 2003 Taylor & Francis Ltd
DOI: 10.1080/13577140310001650330

**Table 1.** *Summary of cases*

| Pt. | Initial tumor | Initial treatment | Prior RT | | Second primary | | Comments |
|---|---|---|---|---|---|---|---|
| | | | Date | Dose (Gy) | Date | Site | |
| 1 | Rectum | RT<br>5-Fluorouracil | 1984 | 50.4 | 1998 (14 years) | Buttock | LTFU |
| 2 | Face | RT<br>Cyclophosphamide<br>azathioprine | 1971<br>1984 | 21<br>45 | 1998<br>(14/27 years) | Maxillary sinus | Doxorubicin +<br>Ifosfamide →<br>Surgery → NED |
| 3 | Larynx | RT 5-Fluorouracil, hydroxyurea | 1989 | 60 | 1998 (9 years) | Trachea | Doxorubicin +<br>dacarbazine → Died |
| | | ALL RT plus antimetabolite | | | 9–27-year interval | | |

LTFU, lost to follow-up; NED, no evidence of disease; RT, radiotherapy; Gy gray.

(a) (b)

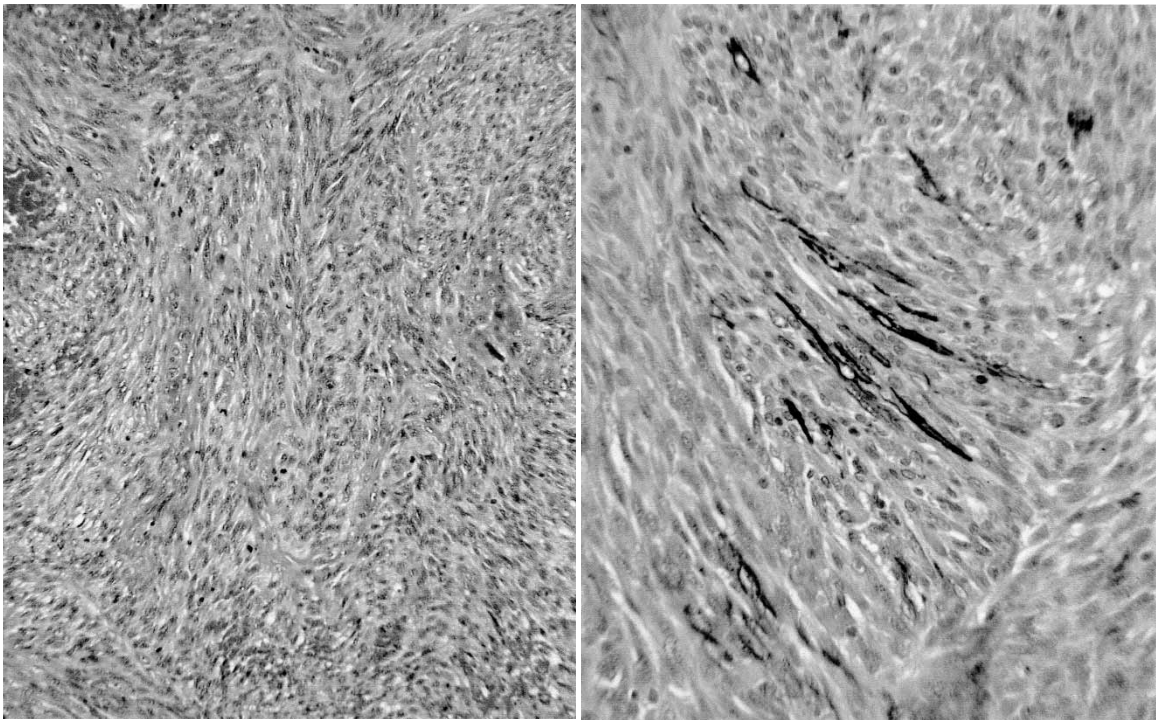

**Fig. 1.** *(a) Low power H&E stain reveals a diffusely elongated spindle cell neoplasm from a 1.5-cm tumor. (b) Representative high power view of immunostain for smooth muscle antigen (SMA). Additional stains for desmin, muscle specific antigen, keratin, HMB45, and S100 were negative.*

(5040 cGy radiation). A solitary liver metastasis was resected in 1990. He developed a spontaneous pneumothorax in September 1998, which recurred after chest tube evacuation. A CXR and subsequent chest CT scan showed bilateral pulmonary nodules. A thoracoscopic biopsy revealed a high-grade leiomyosarcoma (Fig. 1). The patient had a 1-year history of posterior thigh and buttock pain; clinical examination demonstrated a large deep left buttock mass. CT scan demonstrated the primary tumor as an $8 \times 5 \times 4$-cm mass deep to the left gluteal muscles, and a 6-cm ring enhancing collection posterior to the rectum.

*Case 2*

A 60-year-old man had a 30-year history of relapsing ‘midline destructive disease’ and an acoustic neuroma diagnosed in 1995. Aggressive disease over the years resulted in disease-related and surgical loss of nasal and facial bone and cartilage. Extensive past treatment included antibiotics, cyclophosphamide, azathioprine, and multiple surgical procedures. He received radiation to the midline face in 1971 (2100 cGy Cobalt radiation) and again in 1984 (4500 cGy; the exact timing of the azathioprine and cyclophosphamide in relationship to the two courses

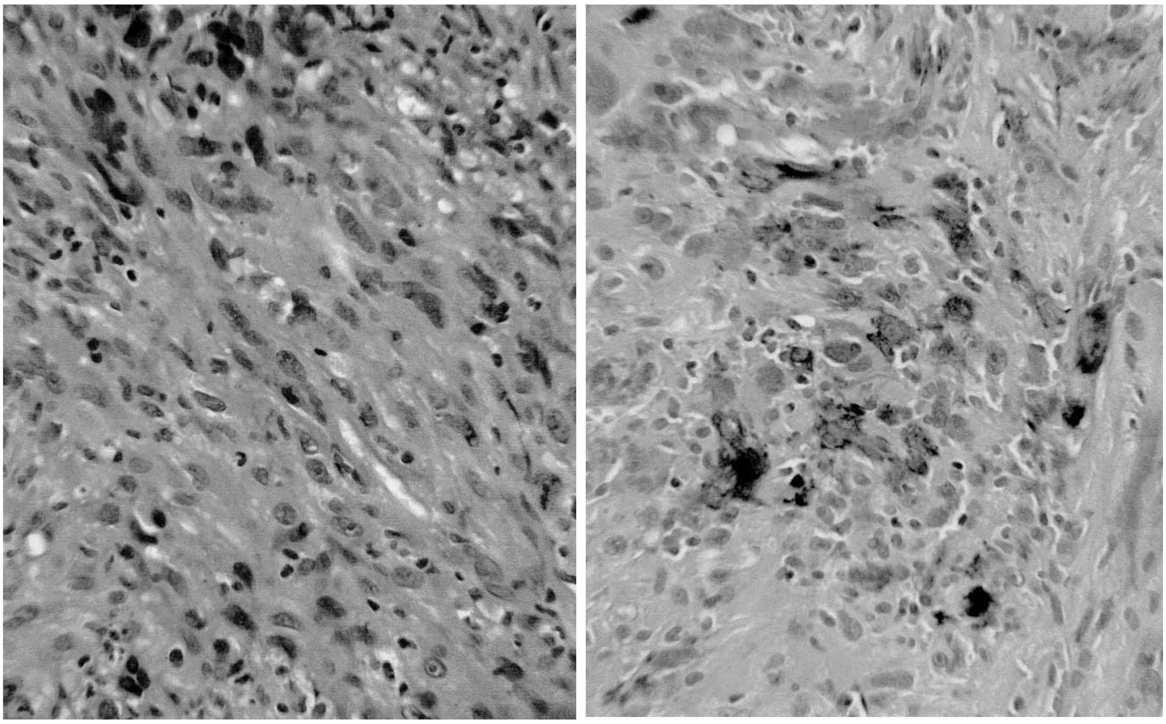

**Fig. 2.** *(a) High power H&E stain reveals a pleomorphic high-grade spindle cell neoplasm. (b) Representative high power view of immunostain for SMA. SMA, as well as muscle specific antigen, is clearly positive in pleomorphic malignant tumor cells. Desmin and Leukocyte common antigen were negative, and S100 and keratin showed occasional positivity. (LMS may occasionally show keratin positivity.)*

of radiation is not known). In June 1998 he noted a small mass beneath the left eye. A work-up included a CT scan of the sinuses, which showed a large mass involving the left face in the area of the left maxillary sinus with extensive destruction of the adjacent bony structures and extension into the ethmoid and sphenoid sinuses. A biopsy in October 1998 revealed a high-grade leiomyosarcoma (Fig. 2). The patient had a dramatic response to three cycles of pre-operative doxorubicin and ifosfamide. Surgical excision of the tumor revealed only microscopic deposits of residual LMS. Two additional cycles of chemotherapy were given, and the patient remained free of disease 1 year later.

*Case 3*

A 76-year-old woman was treated for a T2N0M0 squamous cell carcinoma of the epiglottis in 1989 with laser excision and post-operative infusional 5-FU (800 mg/m$^2$ per day $\times$ 5 days every other week for six cycles), oral hydroxyurea (1000 twice daily for 5.5 days, every other week for six cycles) and radiation (6000 cGy). In October 1998, the patient presented with dysphagia and stridor; direct laryngoscopy and CT scanning revealed a large, partially obstructing tracheal mass (Fig. 3). A tracheal stent was placed, and a biopsy revealed a high-grade leiomyosarcoma (Fig. 4). The mass was unresectable, and she underwent palliative chemotherapy with doxorubicin and dacarbazine in November 1998. She tolerated the chemotherapy well but died within a month of disease progression.

### Discussion

We report three cases of radiation associated LMS occurring 9–14 years following RT (or 9–27, as one patient received two separate courses of RT) in three patients who presented to our clinic in the same month. All had received prior antimetabolite therapy: in one case, 5-fluorouracil; in another azathioprine; and in the third, 5-fluorouracil and hydroxyurea. One of these patients also received the alkylating agent cyclophosphamide.

Clear associations have been made between therapeutic RT for cancer and the development of late second malignancies.[1–7] These associations have also been made for low-dose RT as used in the past for benign conditions such as tonsillar enlargement, menorrhagia, peptic ulcer disease or acne.[8–10] An excess of hematological malignancies and solid tumors occur above that expected for either age-matched controls or disease-matched controls not receiving radiation. Even though soft tissue sarcomas represent only a small percentage of these solid tumors, the excess relative risk at 20 years has been reported to be six- to several hundred-fold greater than expected.[2,6,11] Whereas there appears to be a plateau in the incidence of hematological malignancies at 10 years, the peak solid tumor incidence has not been identified, and series with 15–20 year

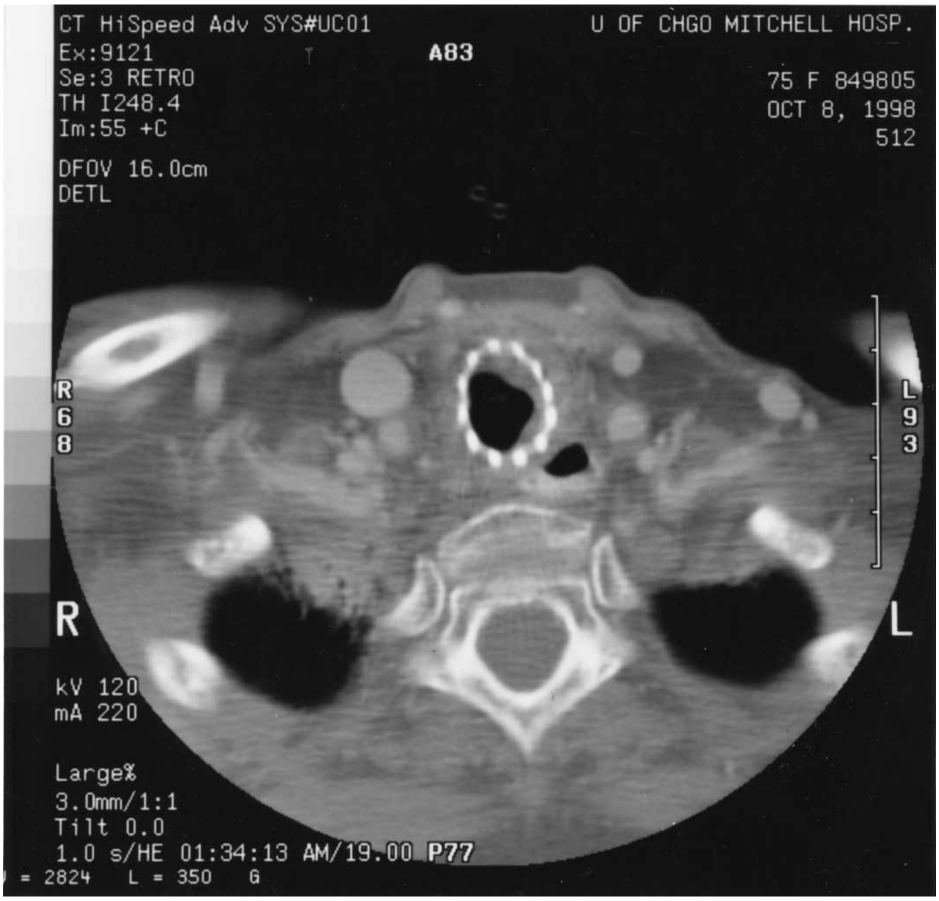

**Fig. 3.** *CT of neck with intravenous contrast shows tracheal mass growing through tracheal ring stent.*

(a) (b)

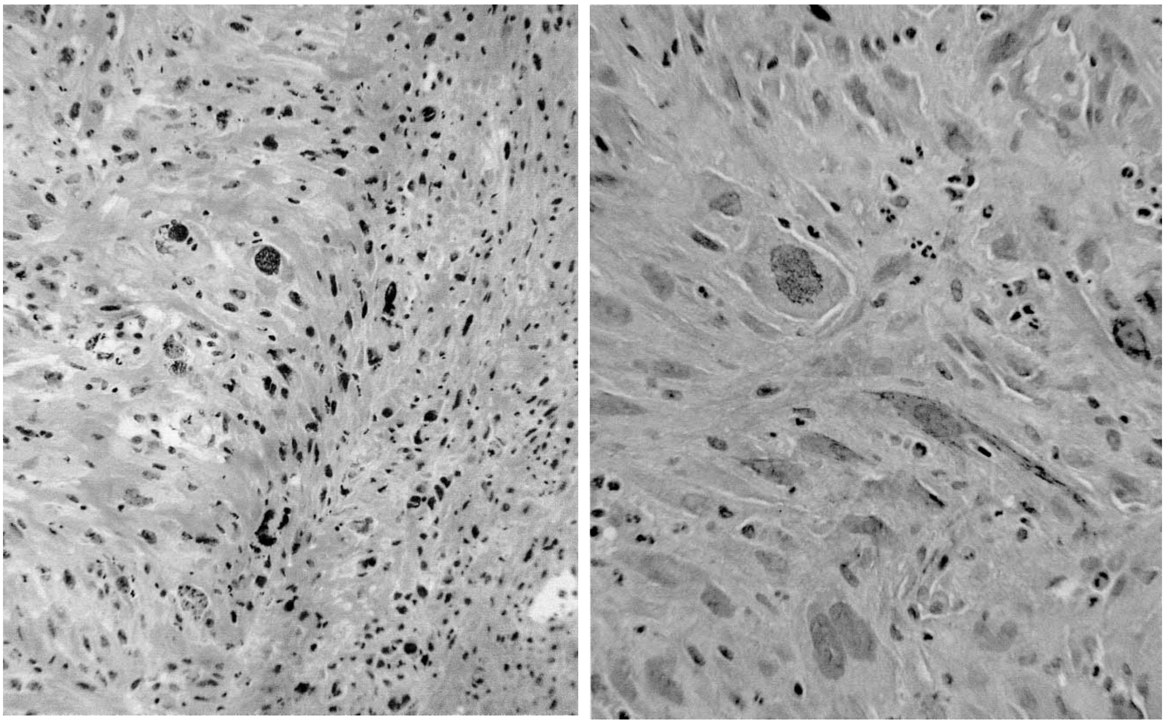

**Fig. 4.** *(a) High power H&E reveals a spindle cell neoplasm with marked nuclear atypia and abnormal mitotic figures. (b) High power SMA stain supports the diagnosis of leiomyosarcoma. Additional stains included vimentin (positive) and keratin (negative).*

follow-up report a total incidence as high as over 20%.[1,3,5,11,12]

Chemotherapy clearly contributes to the development of secondary hematological malignancies but not as clearly to the development of secondary solid tumors. Lung cancer risk is likely increased by chemotherapy,[2] whereas breast cancer risk appears to be more related to RT than chemotherapy.[1,13] In several series reporting post-radiation or radiation-associated sarcomas, the most common subtypes have been angiosarcoma, osteosarcoma, fibrosarcoma, and MFH.[10,15–17] Leoimyosarcoma has not been commonly reported. We were able to find 33 reports of RT associated LMS in the English language literature, comprising 21 single case reports,[18–38] four series of two patients[16,39–41] and one series of four patients.[10] These 33 leiomyosarcomas occurred 6–35 years after therapeutic RT for a variety of benign and malignant diseases. In the largest series, none of the four patients had received chemotherapy, although several other cases have been reported in association with chemotherapy, mostly alkylating agents. The prognosis for patients with post-radiation solid tumors, and sarcomas in particular, is poor.[16,42,43]

All three of our cases of radiation-associated LMS, which occurred 9–27 years after RT, presented to our clinic in the same month. All had received prior antimetabolite therapy: in two cases 5-fluorouracil, and in one case azathioprine. It is possible that antimetabolite therapy leads to mis-incorporation of nucleotides or other faulty DNA repair in the setting of RT induced sublethal damage, allowing for later sarcomagenesis in otherwise vulnerable smooth muscle cells. Prior reports of secondary sarcomas have not reported the association of antimetabolite therapy as a contributory factor. There is, however, a long latency period from radiotherapy to second primary sarcoma, so the rise in excess cases may lag behind any increase in antimetabolite plus radiotherapy use. With the increasing evidence for the use of 5-FU as a radiosensitizer in the treatment of malignancies, such as head and neck cancer, cervical cancer, stomach cancer, rectal cancer, and pancreatic cancer, more such cases may be seen in the future.

### Conclusions

Leiomyosarcoma may rarely occur as a radiation-induced or associated malignancy. In our series of three patients, LMS occurred late after radiotherapy and all had received antimetabolites either concomitant with RT or close to the time of RT. This association has not been previously reported, but there may be a lag time between the increasing use of treatment with RT plus antimetabolite and the development of LMS.

### Acknowledgements

The authors thank Janel Morrow Crittendon for her secretarial assistance with the preparation of the manuscript.

### References

1. van Leuwen FE, Klokman WJ, Hagenbeek A, *et al.* Second cancer risk following Hodgkin's disease: a 20-year follow-up study. *J Clin Oncol* 1994; 12: 312–25.
2. Swerdlow AJ, Douglas AJ, Hudson GV, *et al.* Risk of second primary cancers after Hodgkin's disease by type of treatment: Analysis of 2846 patients in the British National Lymphoma Investigation. *Br Med J* 1992; 304: 1137–43.
3. Tucker MA, Coleman CN, Cox RS, *et al.* Risk of second cancers after treatment Hodgkin's disease. *New Engl J Med* 1988; 318: 76–81.
4. Henry-Amar M. Second cancer after the treatment for Hodgkin's disease: a report from the International Database on Hodgkin's Disease. *Ann Oncol* 1992; 3 (Suppl 4): 117–28.
5. van Leeuwen FE, Klokman WJ, van't Veer MB, *et al.* Long-term risk of second malignancy in survivors of Hodgkin's disease treated during adolescence or young adulthood. *J Clin Oncol* 2000; 18: 487–97.
6. Boivin JF, O'Brien K. Solid cancer risk after treatment of Hodgkin's disease. *Cancer* 1988; 61: 2541–6.
7. Beaty O 3rd, Hudson MM, Greenwald C, *et al.* Subsequent malignancies in children and adolescents after treatment for Hodgkin's disease. *J Clin Oncol* 1995; 13: 603–5.
8. Ron E, Lubin JH, Shore RE, *et al.* Thyroid cancer after exposure to external radiation: A pooled analysis of seven studies. *Radiat Res* 1995; 141: 259–77.
9. Schneider AB, Recant W, Pinsky S, *et al.* Radiation-induced thyroid carcinoma: clinical course and results of therapy in 296 patients. *Ann Intern Med* 1986; 105: 405–12.
10. Mark RJ, Poen J, Tran LM, Fu YS, Selch MT, Parker RG. Postirradiation sarcomas. A single-institution study and review of the literature. *Cancer* 1994; 73: 2653–62.
11. Tucker MA, D'Angio, GJ, Boice, JD Jr, *et al.* Bone sarcomas linked to radiotherapy and chemotherapy in children. *New Engl J Med* 1987; 317: 588–93.
12. Nyandoto P, Muhonen T, Joensuu H. Second cancer among long-term survivors from Hodgkin's disease. *Int J Radiat Oncol Biol Phys* 1998; 42: 373–8.
13. Bhatia S, Robinson LL, Oberlin O, *et al.* Breast cancer and other second neoplasms after childhood Hodgkin's disease. *New Engl J Med* 1996; 334: 745–51.
14. Brenin CM, Small W Jr, Talamonti MS, Gradisher WJ. Radiation-induced sarcoma following treatment of breast cancer. *Cancer Control* 1998; 5: 425–32.
15. Sheppard DG, Libshitz HI. Post-radiation sarcomas: a review of the clinical and imaging features in 63 cases. *Clin Radiol* 2001; 56: 22–9.
16. Murray EM, Werner D, Greeff EA, Taylor DA. Postradiation sarcomas: 20 cases and a literature review. *Int J Radiat Oncol Phys* 1999; 45: 951–61.
17. Sener SF, Feldman JL, Martz CH, *et al.* The spectrum of vascular lesions in the mammary skin, including angiosarcoma, after breast conservation treatment for breast cancer. *J Am Coll Surg* 2001; 193: 22–8.
18. Lee KH, Fiedler P, Passarelli J, Bobrow S. Autoimmune hemolytic anemia associated with postirradiation malignant stromal tumor (leiomyosarcoma) of the jejunum. *Ann Diagn Pathol* 2000; 4: 367–9.
19. Amilibia Cabeza E, Nogues Orpi J, Cruellas Taischik F, *et al.* [Leiomyosarcoma of the larynx]. *Acta Otorrinolaringol Esp* 2000; 51: 85–7.
20. Aoki T, Ozeki Y, Watanabe M, Tanaka S, Isaki H, Terahata S. Development of primary leiomyosarcoma of the sternum postirradiatin: report of a case. *Surg Today* 1998; 28: 1326–8.
21. O'Sullivan SG, Das Narla L, Ferraro E. Primary ovarian leiomyosarcoma in an adolescent following radiation for medulloblastoma. *Pediatr Radiol* 1998; 28: 468–70.
22. Murakami N, Morioka T, Nishio S, *et al.* Radiation-induced leiomyosarcoma of the dura mater: a case report. *No Shinkei Geka* 1997; 25: 1049–53.
23. Stein ME, Leviov M, Drumea K, Goralnik L, Miselevich I, Kuten A. Radiation-induced sarcoma following curative radiotherapy for testicular seminoma: case report and brief review of the literature. *Tumori* 1997; 83: 721–3.
24. Fujii H, Barnes L, Johnson JT, Kapadia SB. Post-radiation primary intranodal leiomyosarcoma. *J Laryngol Otol* 1995; 109: 80–3.
25. Hartley C. Post-irradiation leiomyosarcoma of the maxilla (letter). *J Laryngol Otol* 1993; 107: 1082.
26. Drumea K, Sabo E, Zuckerman E, Naschitz JE. Leiomyosarcoma of the colon in the aftermath of pelvic irradiation for endometrial carcinoma (letter). *Am J Gastroenterol* 1993; 88: 1302.
27. Amichetti M, Boi S. Postirradiation sarcoma in a patient treated for testicular seminoma. *Oncology* 1993; 50: 264–6.
28. Martin-Hirsch DP, Habashi S, Benbow EW, Farrington WT. Post-irradiation leiomyosarcoma of the maxilla. *J Laryngol Otol* 1991; 105: 1068–71.
29. Mihara F, Gupta KL, Kartchner ZA, Kogutt MS, Robinson AE. Leiomyosarcoma after retinoblastoa radiotherapy. *Radiat Med* 1991; 9: 183–4.
30. Yamamura T, Takada A, Higashiyama M, Yoshikawa K, *et al.* Subcutaneous leiomyosarcoma developing in a radiation dermatitis. *Dermatologica* 1991; 183 (2): 154–6.
31. Lalwani AK, Kaplan MJ. Paranasal sinus leiomyosarcoma after cyclophosphamide and irradiation. *Otolaryngol Head Neck Surg* 1990; 103: 1039–42.
32. Stevens GN, Tattersall MH, Stalley P. Leiomyosarcoma following therapeutic irradiation for ankylosing spondylitis. *Br J Radiol* 1990; 63: 730–2.
33. Weiss KS, Zidar BL, Wang S, *et al.* Radiation-induced leiomyosarcoma of the great vessels presenting as superior vena cava syndrome. *Cancer* 1987; 60: 1238–42.
34. Korbi S, Meyer D, Skalli O, Gabbiani G, Kapanci Y, *et al.* Post-irradiation leiomyosarcoma. Case report with immunohistochemical studies. *J Submicrosc Cytol* 1987; 19: 365–9.
35. Schneider K, Dickerhoff R, Bertele RM, *et al.* Malignant gastric sarcoma—diagnosis by ultrasound and endoscopy. *Pediatr Radiol* 1986; 16: 69–70.
36. Font RL, Jurco S 3rd, Brechner RJ, *et al.* Postradiation leiomyosarcoma of the orbit complicating bilateral retinoblastoma. *Arch Ophthamol* 1983; 101: 1557–61.
37. Lynch DF Jr, Herr HW, *et al.* Radiation-induced sarcoma following radiotherapy for testicular tumor. *J Urol* 1981; 126: 845–6.
38. Amendola BE, Amendola MA, McClatchey KD, Miller CH Jr. Radiation-associated sarcoma: a review of 23 patients with postradiation sarcoma over a 50-year period. Am J Clin Oncol 1989; 12: 411–5.
39. Lieber MR, Winans CS, Griem ML, Moossa R, Elner VM, Franklin WA. Sarcomas arising after radiotherapy for peptic ulcer disease. *Dig Dis Sci* 1985; 30: 593–9.
40. Mertens F, Larramendy M, Gustavsson A, *et al.* Radiation associated sarcomas are characterized by complex karyotypes with frequent rearrangements of chromosome 3p. *Cancer Genet Cytogenet* 2000; 116: 89–96.
41. Folberg R, Cleasby G, Flanagan JA, Spencer WH, Zimmerman LE, *et al.* Orbital leiomyosarcoma after radiation therapy for bilateral retinoblastoma. *Arch Ophthalmol* 1983; 101: 1562–5.
42. Weatherby RP, Dahlin DC, Ivins JC, *et al.* Postradiation sarcoma of bone: review of 78 Mayo Clinic cases. *Mayo Clin Proc* 1981; 56: 294–306.
43. Mauch PM, Kalish LA, Marcus KC, *et al.* Second malignancies after treatment for laparotomy staged IA-IIIB Hodgkin's disease: long-term analysis of risk factors and outcome. *Blood* 1996; 87: 3625–32.
